# Choosing and evaluating randomisation methods in clinical trials: a qualitative study

**DOI:** 10.1186/s13063-024-08005-z

**Published:** 2024-03-20

**Authors:** Cydney L. Bruce, Mais Iflaifel, Alan Montgomery, Reuben Ogollah, Kirsty Sprange, Christopher Partlett

**Affiliations:** Nottingham Clinical Trials Unit, School of Medicine, University of Nottingham, Nottingham, UK

**Keywords:** Randomisation, RCTs, Qualitative, Focus groups

## Abstract

**Background:**

There exist many different methods of allocating participants to treatment groups during a randomised controlled trial. Although there is research that explores trial characteristics that are associated with the choice of method, there is still a lot of variety in practice not explained. This study used qualitative methods to explore more deeply the motivations behind researchers’ choice of randomisation, and which features of the method they use to evaluate the performance of these methods.

**Methods:**

Data was collected from online focus groups with various stakeholders involved in the randomisation process. Focus groups were recorded and then transcribed verbatim. A thematic analysis was used to analyse the transcripts.

**Results:**

Twenty-five participants from twenty clinical trials units across the UK were recruited to take part in one of four focus groups. Four main themes were identified: how randomisation methods are selected; researchers’ opinions of the different methods; which features of the method are desirable and ways to measure method features.

Most researchers agree that the randomisation method should be selected based on key trial characteristics; however, for many, a unit standard is in place.

Opinions of methods were varied with some participants favouring stratified blocks and others favouring minimisation. This was generally due to researchers’ perception of the effect these methods had on balance and predictability.

Generally, predictability was considered more important than balance as adjustments cannot be made for it; however, most researchers felt that the importance of these two methods was dependent on the design of the study.

Balance is usually evaluated by tabulating variables by treatment arm and looking for perceived imbalances, predictability was generally considered much harder to measure, partly due to differing definitions.

**Conclusion:**

There is a wide variety in practice on how randomisation methods are selected and researcher’s opinions on methods. The difference in practice observed when looking at randomisation method selection can be explained by a difference in unit practice, and also by a difference in researchers prioritisation of balance and predictability. The findings of this study show a need for more guidance on randomisation method selection.

**Supplementary Information:**

The online version contains supplementary material available at 10.1186/s13063-024-08005-z.

## Introduction

Randomisation is considered the gold standard for allocating interventions in clinical trials [1]. It allows researchers to control for selection bias, which can result in systematic differences in the characteristics of participants being compared in each treatment group. In addition, some allocation methods can be used to improve balance between randomised groups with respect to certain characteristics. There are various methods to achieve random allocations, each with different benefits and disadvantages [2].

Simple, or unrestricted, randomisation, for example, creates an entirely unpredictable sequence; however, this comes at the potential loss of balance of participant numbers and characteristics in each group. Restricted allocation methods, such as minimisation can ensure better balance with respect to key characteristics, but this comes at the cost of being more predictable [2, 3].

The International Conference on Harmonization (ICH) E9 guideline “Statistical Principles for Clinical Trials” [4] developed by the European Medicines Agency (EMA) have given advice on randomisation selection as follows:

- Unrestricted randomisation is an acceptable approach, but using blocks of an appropriate length has additional advantages.
- Separate randomisation schemes should be used in multicentre trials. Stratification can also be valuable for important prognostic factors measured at baseline to promote balance. However, more than two or three stratification factors are rarely necessary.
- Deterministic dynamic allocation methods should be avoided. They can be used with an appropriate random element and the potential impact on analysis should be considered.

There are some scenarios in which an allocation method may not be adequate. Simple randomisation can be susceptible to chronological bias and confounding, making the method potentially inadequate in small sample sizes with known confounders [5]. Permuted blocks have been shown to lead to more predictable sequences, even with varying block sizes, leaving the method susceptible to selection bias in unblinded studies [6]. Failure to select a randomisation method that is compatible with the trial design can lead to inefficiency or, worse, erode confidence in the trial conclusions.

Previous research shows there is little association between trial characteristics, other than the size of the trial, and choice of allocation method used in randomised trials [7, 8]. How researchers choose how to allocate participants in randomised trials remains largely unexplained.

The aims of this research were twofold. The first was to investigate the motivations behind researchers' selection of randomisation methods, and the second was to identify the key features to consider when evaluating how different allocation methods perform.

## Methods

Qualitative focus groups were facilitated by one researcher (CB) with support from two additional researchers (CP and RO). All three researchers have a statistical background. Two additional researchers (KS) and (MI) who have experience in qualitative methods were consulted on the design of the study and on the analysis method used.

Initially, four focus groups containing 4–8 researchers were planned, but it was decided that all interested participants would be included.

### Recruitment

We aimed to recruit from three main stakeholder groups: Statisticians involved in the randomisation process, programmers who designed randomisation algorithms, and other members of clinical trials teams with experience of selecting allocation methods.

A sample of researchers from the above groups was recruited from the UK Clinical Research Collaboration (UKCRC) statistics, information systems and trial management working groups, and from the Trials Methodology Research Partnership (TMRP) statistical analysis and adaptive design working groups. The latter group was included with the aim of finding researchers experienced in less commonly used randomisation methods.

An invitation email was circulated via group membership lists containing a link to a short survey. This survey was used to collect contact information for interested researchers, as well as additional information on job role and any relevant additional experience such as having worked in the pharmaceutical industry or having been part of an oversight committee. Those who completed this survey were approached using a participant information sheet (Additional file 1). The survey data was used to create more balanced groups in terms of job role and level.

### Data collection

The focus groups were conducted remotely using Microsoft Teams, with video and audio recordings, to be transcribed. At the beginning of each focus group consent of participants was verbally confirmed before recording began. A topic guide, developed and further revised following an initial pilot focus group, was used to shape the discussion. Following the pilot focus group, only small changes were made to the topic guide, hence it was felt the pilot focus group did not significantly differ from other groups and that the data collected was just as valid to the project as other focus groups. Participants were asked to draw on all previous experiences when considering the discussed topics.

The topic guide included five key sections (details of which are available in Additional file 2):

1. Randomisation method selection
2. Randomisation method opinions
3. Which method features are important?
4. Why are method features important?
5. How are method features measured/quantified?

When designing the topic guide for this study, definitions for the terms ‘balance’ and ‘predictability’ were presented to participants to ensure they used the same definitions consistently, and they were asked to consider these when responding to questions. These definitions are given in Table 1.

**Table 1:** Topic guide definitions of balance and predictability

Balance—“Balance is concerned with making sure groups are similar, both in size and division of characteristics.”
Predictability—“Given information about previous allocations, how easily can a recruiter guess the next allocation?”

During the focus groups those facilitating also used prompts in addition to the interview guide to encourage discussion or to follow-up on participant responses.

### Analysis

Automatic transcripts were produced directly from the video recording, which were reviewed and corrected by CB. All transcripts were then open coded by CB. The analysis included a deductive approach to identify themes based on the interview schedule topics as well as inductive to identify new emerging themes. Qualitative data management software, NVivo 12, was used to store and manage the data and categorise important themes from the data. A framework model was used to organise and chart the data [9]. This study followed the consolidated criteria for reporting qualitative research (COREQ) guidelines [10] (see Additional file 3). Transcripts are stored in a secure location with access only available to the study team. Quotes used have been anonymised.

Subthemes were identified from analysing the focus group transcripts that aligned with the 4 main questions presented to participants in the topic guide and are presented in the results.

## Results

Four focus groups were undertaken from May 2022 to June 2022. Thirty-one researchers responded to the initial recruitment email and were subsequently contacted with a follow-up invitation email, information sheet and consent form to take part in a focus group., Of those, 25 researchers from 20 different UK Clinical Trials Units (CTUs) responded and agreed to take part. The other 6 did not respond to the invitation email.

Focus groups were made up of 3 mixed groups, and 1 group containing only statisticians. Table 2 summarises the roles and CTUs in each of the focus groups.

**Table 2:** The roles of participants and the number of units they came from summarised by focus group

Role	Pilot FG	FG 1	FG 2	FG 3
	**(** ***n*** ** = 6)**	**(** ***n*** ** = 5)**	**(** ***n*** ** = 9)**	**(** ***n*** ** = 5)**
**Statisticians**	4	4	7	5
**IT/programmers**	2	0	2	0
**Other**	0	1	0	0
**Number of CTUs represented**	4	5	8	5

Identified subthemes fall under the following 4 headings:

- *Question 1* — selection of randomisation method
- *Question 2* — participants opinions of the different randomisation methods
- *Question 3* — desirable features of a randomisation method
- *Question 4* — measuring/quantifying features of a randomisation method

Where numbers of participants are provided in the findings these numbers do not represent a proportion of the total number of participants, they represent those participants only who expressed a particular view. Not all participants may have commented on every question.

### Question 1 — selection of randomisation method

Two main factors for selecting a randomisation method were identified, unit standard and trial design as shown in Table 3. Ten participants (from 9 CTUs) discussed how their unit had a preferred method, sometimes due to implementation costs and unit expertise (further supporting quotation is listed in Additional file 4: Table S1).

…the default position now in [unit] is minimisation and therefore you know, it’s almost like we justify not minimising … (Statistician 1).

**Table 3:** Summary of randomisation method selection themes

Sub-theme ^a^	Description	FG 1^b^ (*n* = 6)	FG 2^b^ (*n* = 5)	FG 3^b^ (*n* = 9)	FG 4^b^ (*n* = 5)	Total^b^ (*n* = 25)	Illustrative quote
**Unit standard**	The unit might have a specific method that would be the default – deviation from this is either rare or needs to be clearly justified	**4**	**2**	**1**	**3**	**10**	“…there’s like a default institutional sort of preference. The last place I worked that was also a default institutional preference as well for minimisation.” **(Programmer 3)**
**Expertise**	Statisticians or programmers may be more familiar with a specific method making it easier to implement	**1**	**3**	**3**	**3**	**10**	“We have like an in-house system that we use so it doesn't really make any difference. Obviously at the start like when it was first created there was, you know, how are we going to design minimisation.” **(Statistician 8)**
**Cost and time**	Trial set-up often has limited money and time, so resource constraints may limit the available methodology	**2**	**1**	**2**	**3**	**8**	“I guess there might be an element of cost that we need to consider because obviously minimisation would cost more” **(Statistician 13)**
**Trial design**	The design of a trial affects how well each of the methods perform	**3**	**2**	**5**	**3**	**13**	“You look at the trial design and talk to the investigators” **(Statistician 7)**
Sample size	With smaller sample sizes the affect of imbalance may be greater so more restrictive methods may be preferred	3	3	1	2	9	“You’re going to base it on your sample size. And also like, the number of factors that you wanted to cater for” **(Statistician 15)**
Number of variables	The number of important prognostic variables you wish to include may influence this, e.g. stratification may not be able to handle a large number of variables with many strata	3	2	3	1	9	“I tend to question protocols when I see more than two variables and the word stratification, I think why are you doing this basically” **(Programmer 2)**
**External influence**	The choice of randomisation method may be influenced by individuals not a member of the trial team	2	3	2	0	7	“But what matters is getting the publication out. And being able to show that you did something that was unpredictable and ended up with the balanced allocation in relation to key important variables. It’s the perception to the outside world, as much as anything.” (Statistician 9)

^a^Note that categories are not mutually exclusive. Some participants who discussed having a unit standard also discussed a personal view that trial design should be considered

^b^Each cell denotes the number of participants who mentioned this theme being important to consider during focus groups

Alternatively, those who did not report having a unit standard used the trial design to determine the method. The trial sample size and the number of variables used in the randomisation seemed to be the most important design features to consider. External influences were also attributed to informing this decision by 7 participants, listing the Medicines and Healthcare products Regulatory Agency (MHRA) and other regulatory bodies, the funders and publishers.

Now fifteen years ago, the European Medicines Agency was anti minimisation. (Statistician 11).

Aiming to comply with European Medicines Agency (EMA) guidance could lead researchers to avoid minimisation, whilst reviewers at the grant application stage may also affect the final choice of method, or randomisation variables included.

Some participants indicated that randomisation was outsourced to another company, whilst others indicated that their randomisation was performed in-house, which led to internal expertise playing a greater role in method selection.

When discussing who was involved in the selection process of randomisation methods, many participants indicated the need for more collaboration in teams; however, the statistician and chief investigator (CI) were named as being involved in the decision.

I’ve tried to engage other members of trial teams when designing trials so that designing randomisation is very much a multidisciplinary process (Statistician 2).

Finally, a few participants noted that their beliefs of how these methods should be selected did not always align with practice.

I would echo that disparity between what happens in practice and what happens in reality. (Statistician 2).

Some of the participants who stated that there was a unit standard did feel that more thought should be put into the selection process, but cited time, money, and expertise constraints as to why this did not happen.

### Question 2 — participant opinions of the different randomisation methods

Most participants did not have strong opinions with respect to any of the randomisation methods, but instead, they felt that a good reason is needed to select any of the methods.

I don’t think a particular default position is healthy actually. I think you need good reasons for choosing either method. (Statistician 3).

The discussion predominantly covered three main randomisation methods; (i) simple, (ii) stratified block, and (iii) minimisation, and each of these are discussed in more detail in this section. A glossary of each of these terms is shown in Table 4.

**Table 4:** Summary of concerns regarding randomisation methodology

Method	Definition
Simple randomisation	Each allocation is determined entirely randomly. In a two-arm trial with 1:1 allocation this is equivalent to flipping a coin for each allocation
Stratified block randomisation	Stratified randomisation involves separate lists being produced for different factors of a variable to obtain balance. The lists are typically compromised of ‘blocks’ of varying sizes that ensure the target allocation ratio is maintained For example, in a two-arm trial with 1:1 allocation to groups A and B, a block of size 6 would be made up of a sequence of three As and three Bs in a random order
Minimisation	Minimisation involves an algorithm that considers characteristics of previously recruited participants and aims to allocate the next participant so as to minimise the imbalance in these factors between groups. This will typically involve a “random element” where the allocation will, with a predetermined probability, allocate at random instead

#### Simple randomisation

Although simple randomisation was widely accepted as an appropriate randomisation method for large sample sizes, very few researchers actually reported using this method, with many shying away, feeling that some form of restriction was needed to ensure some balance.

…that’s probably the reason why I’ve never used simple randomisation. I’d just being too worried that it might go adrift. (Statistician 4).

Multiple statisticians mentioned that although they felt the method was valid in studies with large sample sizes, other members of the trial team were not keen on a method that uses no restriction. Programmers commented that it was much more difficult to monitor the method.

Simple randomisation. It’s given me more sleepless nights than any other randomisation because we’ve had some horrendous balance. So one of the arms after 24 randomisations, 20, had gone into one arm, and you just if you were tossing a coin, you think there was something rather biased about it, and there’s no way to check. (Programmer 1).

#### Stratified blocks vs minimisation

In most cases, selection of a randomisation method was regarded as a choice between 2 methods: stratified block randomisation and minimisation, with few considering other methods beyond these.

##### Stratified blocks

Most participants agreed that stratified block randomisation is a valid and useful method of randomisation. Their views reflected the fact that blocks ensured balanced numbers in each group whilst strata can ensure balance with respect to a few characteristics; also, by using randomly permuted blocks, predictability can be greatly reduced.

The smaller the design, maybe you might consider minimisation, but generally for a reasonable size, go for stratified block randomisation. (Statistician 7).

Well, in terms of predictability, having more strata makes it harder for a site to be able to predict what’s coming next. Mixed block size is always help. If they don’t know how big the block is, they can’t see when the end’s coming. (Statistician 9).

Some participants however identified reservations around employing this method when there are a large number of strata. For example, including centre in the randomisation could lead to more imbalance as some blocks may never be completed.

…you can’t tell how the lists for the different stratification groups are going to get filled up so and with variable block sizes, you could, you know, quite unfortunately, go to a situation where you get actually a huge imbalance and that would be perfectly following the lists. (Programmer 1).

Others questioned the predictability of this method, especially when centre is included in the randomisation. When asked about when they would or would not feel comfortable to use this method, most participants agreed they would not include more than 3 variables with a small number of categories in stratified block randomisation.

Yeah, beyond three [stratification variables], I’d probably go for minimisation… (Statistician 9).

##### Minimisation

Most participants agreed that minimisation was most appropriate when a trial has ‘too many’ variables for stratified blocks or should be used with a small sample size. Those who favoured the method appreciated the ability to be able to include a multitude of factors as well as its perceived unpredictable nature in such situations.

If you’ve got a small number of 1 or 2 maybe 3 maximum binary factors that I think stratified probably takes it. If it's anything more than that, minimisation is probably essential… (Statistician 3).

However, those with reservations felt that the method could be overcomplicating the randomisation (allowing clinicians to list more prognostic values to balance on), and some expressed concerns over predictability.

I’ve had clinicians who want to balance on hundreds of factors because they think it may affect outcomes with or without treatment (Statistician 5).

#### Other methods

Most discussions focus on the main methods mentioned above, however, there was discussion of other methods.

Adaptive methods were discussed in three out of the four focus groups; however, some of our participants when asked were unaware of the methods and needed further details. Only one of the participants (statistician) had implemented these methods previously.

A lack of awareness may have contributed to their slow uptake. It was also discussed that another barrier is the fact that frequently used randomisation providers such as Sealed Envelope do not offer adaptive methods as standard. This means that to implement an adaptive method, researchers must either incur further costs for each alteration to the allocation ratio or design these methods in-house which can be very computationally intensive.

These barriers, rather than any fundamental opposition to the methodology, seemed to drive the lack of consideration given to these methods.

So why would you choose a design that leaves you with a lot of extra work on your back? That is not probably costed in any grant (Statistician 12).

Additionally, in one focus group, Atkinson’s method [11] was discussed as a better alternative to minimisation, however, it was also noted that this method requires full specification of the model beforehand, which can be very difficult to do.

Senn [Stephen Senn, Statistician] argues that you should use Atkinsons method, which requires you to know the model, so you have to fully specify the model before you start … you might not know all these things. (Statistician 3).

#### A model for method selection

By synthesising the general opinion of researchers, we have found a general method that participants who considered trial characteristics would follow, shown in Table 5.

**Table 5:** Model for randomisation method selection most considered

Characteristic		Method
Sample size	> 100/ > 1000	Simple
	< 100/ < 1000	Consider restrictive methods
Number of variables	< 3	Block stratified
	> 3	Minimisation

### Question 3 — which features of a randomisation method are desirable

#### Balance vs unpredictability

Discussion largely focussed on the trade-off between two desirable features of a randomisation method, balance and unpredictability. Although most participants stated that importance was dependent on the design of the trial, many when asked still indicated a preference. From Table 6 below, a clear preference for unpredictability can be seen. This is illustrated by comments made by multiple focus group members:

…there’s obviously ways for us to prepare analysis for imbalance, but it’s much harder to address the potential bias from the allocation concealment, or lack of, from predictability. (Statistician 6).

You’re trading off balance, which you can quantify against, for me, a far more important predictability which unless you go out and do field qualitative work, you will never find out. (Statistician 11).

**Table 6:** Participant opinions of whether balance or Unpredictability is most important

Feature	Number of participants who discussed a preference
Balance	1
Unpredictability	7
Both	1
Neither	2
Did not specify a preference	14

A few focus group members discussed that imbalance can be handled in the analysis, which seemed to explain the elevated importance of achieving unpredictability. Instead, balance was considered a more practical issue, with the importance being more focused on balancing intervention delivery within recruiting sites, or considerations of safety outcomes. For example, by balancing on site, managing investigational medicinal product stock was easier as an equal number of each treatment could be delivered to each site. Balance in terms of participant characteristics also aided analysis/interpretation of safety data, which could not be dealt with in the analysis. Balance was considered a more important issue in trials with a lower number of participants.

…sometimes drug supply means that you’re forced to stratify by site, not through any particular biological reason or scientific reason, It’s just practicality. (Statistician 7).

Predictability was considered important to reduce risk of selection bias. Whilst many researchers felt that this issue was less important when trials were blinded, a small number of researchers did caution that a blinded trial did not always mean that predictability was protected and that clinicians may be aware of allocation even in a blinded trial due to effects of the treatment on participants or depletion of stock at sites.

so there is the potential for some predictability in that you may see the depletion of stock in that arm at a greater rate than the other arm. (Programmer 2).

There was also a discussion about how balance is often not done for statistical reasons as much as to appease other members of a trials team. Clinicians may feel uncomfortable not balancing on something, and a few participants discussed how balancing may be for publication, as publishers may be uncomfortable seeing a trial without some form of balancing.

I think often it makes clinicians feel more comfortable to balance on things. (Statistician 8).

#### The definition of predictability

An alternative definition for predictability was identified in the first focus group compared to the one we had presented in the topic guide. This definition centred around how often recruiters try to deliberately subvert randomisation (related quotes are presented in Additional file 4: Table S3) and seemed to change participants’ view on the importance of predictability.

Participants that used our definition tended to consider predictability more important than those who defined predictability around how much recruiters are trying to subvert randomisation. This was due to a perceived lack of attempts to subvert randomisation.

…many of the trials I work in, which are often in like trauma situations or whatever; they don’t have time to guess what the next treatment is. (Statistician 8).

#### Other features

Three other logistical issues found to impact on the decision-making process are also presented in Table 7.

**Table 7:** A summary of logistical issues relating to methods

Feature	Description	Number of participants who discussed this
Time and money	The resource cost of implementing the method. Trial funding can be limited and so this may exclude more costly randomisation methods	4
Programming expertise	The programming expertise required to implement the method. For example, dynamic methods (such as minimisation) require integration with the trial database	6
Statistical expertise	The statistical expertise required to implement the method. For example, implementation of adaptive randomisation methods requires specialist statistical knowledge	6

### Question 4 — how to measure/quantify features of a randomisation method

Participants discussed whether they measured and monitored the performance of randomisation methods, and if so, how they did it. Again, discussion largely focused on balance and predictability. Participants generally were comfortable with the idea of measuring and monitoring balance, while monitoring predictability was a much more challenging issue.

#### Balance

While many participants stated they did not directly check balance, researchers often confirm balance throughout the trial due to production of baseline characteristic tables in interim reports for the data monitoring committee. The baseline characteristics table allows researchers to confirm that there are similar numbers of participants per treatment group, and to check stratification or minimisation variables are balanced. One participant, however, discussed the difficulties of quantifying balance:

I think, balance might be the tougher one to quantify, because are you going to measure it on relative scales or absolute scales? Are you measuring differences? Are you measuring each variable in your table one in isolation, or are you assuming they’re correlated and looking at some multivariate measure? (Statistician 10).

#### Predictability

When discussing predictability, many participants stated that they did not measure the effect of predictability throughout the trial as this was too difficult to measure. Again, the differing definitions of predictability did bring different opinions here, with those using our definition (Table 1) suggesting the need to decide on rules that could reasonably be used by recruiters and testing how often a recruiter would be correct with this pattern.

…the horse I will back is the one that’s had the less allocations. So if you’ve seen seven of A and six of B, I’m accepting this only works if it’s open label, you will always get more than 50% accuracy by saying, well, the next one is more likely to be B. (Statistician 11).

Those whose definition of predictability hinged on how often researchers attempted to subvert the randomisation acknowledged that this would be very difficult to measure.

## Discussion

The aim of this study was to identify researcher motivations behind differences in practice in randomisation method selection, and to identify key method features that factor into these choices. This study found variation in practice, both between CTUs and sometimes within a CTU. Some CTUs had a clear method preference set as an institutional standard, at which point infrastructure built around this method can make using another method more difficult. Others decide the method based on trial design. In both cases, opinions on method performance came down to opinions of the importance of balance and predictability, and the perceived effect that each method had on these features.

### Question 1 — selection of randomisation method

Two main approaches were identified in focus groups which influenced methods selection, either a unit standard or based on the characteristics of the trial. A previous review [7], found that sample size and the number of centres seemed to be associated with choice of randomisation method, and this is in agreement with focus group findings.

However, many of the participants who stated they had a standard choice of method still acknowledged the importance of the trial design in how appropriate a randomisation method is. The general approach to method selection that seemed to be suggested by participants was in line with the EMA E9 guidance on randomisation method selection [4]. Considering that these views did not appear to reflect the participant’s practice, it is possible that researcher’s opinions come from knowing this guidance.

### Question 2 — participant opinions of the different randomisation methods

Within the literature, some researchers regard simple randomisation as the only true randomisation method and believe it should be more widely implemented [12, 13] whilst others regard minimisation as the gold standard for obtaining balanced sequences [14].

Within our focus groups, whilst participants acknowledged the usefulness of simple randomisation, many did not use the method as they were concerned about the risk of imbalance. Even though studies showed that above a sample size of 200 these methods are valid and unlikely to experience issues with balance, [15–17] researchers in our groups were still reluctant to use these methods due to risk of imbalance, or perceived concerns from clinical staff.

Stratified randomisation, when considering the EMA’s guidance [4], is the preferred method, with the statement that more than two or three randomisation variables are usually not necessary. Focus group participants generally agreed up to three variables, but for more than this would use an alternative method such as minimisation.

There was a notable split in preferences between stratified block randomisation and minimisation relating to differing opinions of the effect methods had on balance and predictability. This suggests there is much needed research into the effect each of these methods has on predictability and balance measured in a multitude of ways in order to reassure researchers of the value of both methods and to hopefully lead researchers to the most efficient method based on the design on the trial.

### Question 3 — desirable features of a randomisation method

Balance and predictability are long debated to be the two most important considerations when selecting a randomisation method [18, 19]. Balanced groups can avoid issues in comparisons and make analysis easier, but come at the cost of more predictable sequences, which could lead to selection bias. The perceived importance of predictability was dependent on how researchers defined it.

The differing predictability definitions was a very unexpected outcome of the focus groups, being mentioned in 3 of 4 focus groups, suggesting a need for greater consideration on how both balance and predictability are defined and measured. Additional consideration should be given to logistical issues.

### Question 4 — measuring/quantifying features of a randomisation method

Although there is some research into methods to measure balance and predictability [20–22] this research does not appear to be used in practice among our study participants. Within focus groups, we found researchers had opinions on how methods would perform with respect to these features but did not implement any formal tests.

The differing definitions of predictability gave more context to the issues of measuring predictability. Participants who used our definition of predictability did have ways to measure this, although only theoretically. Those who felt that predictability should be defined by how often recruiters try to make these predictions felt that measuring this would be incredibly difficult, perhaps even impossible.

Previous research suggests that clinicians are not trying to subvert a randomisation sequence [18], suggesting less importance of predictability in this sense, the opinion of which was backed up by many of our focus group participants. However, this research relied on asking recruiters if they attempted to predict. When stating reasons recruiters did not try to predict allocations, one of these was that they were “aware this was wrong” which would suggest they may not want to answer this question honestly.

A case study previously looked at a trial where randomisation appeared to go wrong. Although they could not identify specific instances of misallocation, the study suggests inadequate allocation concealment did lead to allocation subversion [23] suggesting potentially a higher risk of subversion than reported.

Previous research in this area is however quite dated and it might be that understanding of the need for randomised trials and equipoise has increased, suggesting more research is needed to properly evaluate how often recruiters do attempt to subvert randomisations.

### Strengths and limitations

This study benefits from having recruited researchers from a wide variety of institutions and with varying levels of experience and different roles. During recruitment, there was a push to include more members of trial management groups, but there was no additional uptake. Having only 1 participant who was not a statistician or programmer, this may reflect that these 2 roles play the biggest part in the randomisation method selection process and that other roles were unable to contribute to the group, rather than us missing a key stakeholder.

A limitation of this study is the fact that coding was only completed by one researcher, CB. We acknowledge that this does have the potential to introduce bias, however, feel that the effect would be limited. Focus groups were always attended by at least 2 researchers working on the project. During focus groups, we encouraged open and honest discussions, which is evidenced by the contrasting opinions presented in the data and in the analysis.

## Conclusions

This study found there was a variation in practice in the selection and implementation of randomisation methodology. Whilst some researchers had preferences towards specific methods, many admitted that greater consideration should be given on how these methods are selected.

One of the biggest findings of note is that in many situations, the researcher’s views did not necessarily align with their current practice and many researchers had differing views of the of the effect methods had on balance and predictability. This highlights the need for greater investigation into randomisation method performance and the need for guidance to ease researchers when making these decisions.

**Table:** 

**Main findings**
1. Randomisation methods are chosen either based on method preference or based on trial characteristics including sample size
2. Researcher’s opinions of methods are in line with EMA’s guidance – even when not followed in practice
3. Balance and predictability are considered the most important features of a randomisation method, although opinions on their relative importance are varied
4. Although researchers consider balance and predictability important in trials, they are not measuring these features in practice

### Supplementary Information

**Additional file 1.**

**Additional file 2.**

**Additional file 3.**

**Additional file 4: Table S1.** Quotes on institutional standards. **Table S2.** Additional study design features mentioned by researchers. **Table S3.** Quotes around the definition of predictability.

## Data Availability

To protect the identity of focus group participants, source data is not available to request.
