# Supplementary material for: Choosing and evaluating randomisation methods in clinical trials: a qualitative study
Source: Trials. 2024 Mar 20;25:199. doi: 10.1186/s13063-024-08005-z (PMC10953118; doi:10.1186/s13063-024-08005-z)
Supplement: Supplementary file 1 — Additional file 1. [file 13063_2024_8005_MOESM1_ESM.docx]

**Participant Information Sheet**

**Study title: Methods of randomisation**

We would like to invite you to take part in a focus group to discuss your experiences and opinions on the practice of selecting randomisation methodology for a randomised controlled trial. This research will be used to gain a better understanding of the motivators behind randomisation method selection.

**What is the purpose of this study?**

There exist many methods for randomly allocating participants to trials and each method has different benefits and drawbacks. There are varying opinions on which method is considered the most effective. With these focus groups we are aiming to identify two things:

1. How researchers currently select a randomisation method
2. The features of a randomisation method that are most important to assess the methods performance.

This information will be incorporated along with other research to create evidence-based guidance for researchers on which randomisation method is most effective for a given study design.

**What does the study involve?**

We wish to explore the experiences and opinions in those who have worked on clinical trials and have experience of selecting a randomisation method. This will primarily be statisticians, database developers, and programmers, but we are also interested to hear the opinions of other research team members that may be involved in making decisions of what randomisation method to use (such as Chief Investigators or Trial Managers).

If you agree to participate, you will be asked to attend a focus group.

This is an organised discussion with a group of people to gain their views and experience in a specific topic, which in our study will be the experiences, opinions and ideas surrounding randomisation method selection. You will join a focus group with other participants. The researcher(s) will contact you to arrange a suitable time for a focus group. The focus group will be conducted using the Microsoft Teams Platform, will be video and audio recorded and will last approximately 90 minutes.

Questions will be open-ended allowing you the chance to raise the issues that you feel are most important.

**Do I have to take part?**

Taking part is entirely voluntary but if you do take part you can withdraw from the study at any time. If you decide to participate, you will be asked to give consent.

**Are there benefits to me in taking part in this study?**

Sharing your experiences and ideas will help us to develop a guidance document on the most effective randomisation method for a given study design.

**Will what I say be confidential?**

Yes. We will follow ethical and legal practice and all information about you will be handled in confidence. The data will be anonymised by using codes on focus group transcripts. Any quotes used in the research, e.g. publications, presentations at conferences or seminars will use non-identifiable codes rather than the participant’s name. Electronic data will be password-protected and saved on the University of Nottingham’s server.

University of Nottingham will keep identifiable information about you for seven years after the study has finished.

The research team will use your name, email, and telephone number to contact you about the research study. The only people in the University of Nottingham who will have access to information that identifies you will be the research team who need to contact you to arrange for a focus group.

**What will happen to the results of the study?**

Once the focus groups have been transcribed and analysed, the findings will be published in scientific journals and presented in conferences, seminars and workshops. This will add to the information of what features should be considered and monitored when selecting a randomisation method.

This research will then feed into the next stages of the PhD research, where the features identified will be quantitatively measured (where possible) and will be developed into recommendations on which randomisation method performs best for given study designs. Quotes from focus may be used but will be anonymised.

**Who is organising and funding the study?**

The study is being led by researchers from the Nottingham Clinical Trials Unit, University of Nottingham, and is part of a PhD funded by the Nottingham Clinical Trials Unit in association with the Trials Methodology Research Partnership.

**Where can I get more information**

If you have any additional questions or queries, please contact

- Cydney Bruce. Email: [cydney.bruce@nottingham.ac.uk](mailto:cydney.bruce1@nottingham.ac.uk)
