# Supplementary material for: Choosing and evaluating randomisation methods in clinical trials: a qualitative study
Source: Trials. 2024 Mar 20;25:199. doi: 10.1186/s13063-024-08005-z (PMC10953118; doi:10.1186/s13063-024-08005-z)
Supplement: Supplementary file 2 — Additional file 2. [file 13063_2024_8005_MOESM2_ESM.docx]

**Topic Guide**

1. **Welcome**

- My name is Cydney Bruce, and I am a statistician from Nottingham Clinical Trials Unit, University of Nottingham. As part of my PhD, I am looking into how researchers decide upon appropriate randomisation methodology for their study.
- Thank you for agreeing to participate. There are two main aims to this focus group. Firstly, to identify how the decision of which randomisation method to use is currently made when designing randomised controlled trials.
- And secondly which features of a randomisation method should be considered when comparing the effectiveness of the methods.
- We are keen to get your opinions and learn from you, and your input will help us to develop evidence-based guidance and recommendations for randomisation method selection.

1. **Focus group/interview ground rules**

- Before we start, I would like to mention a few things about this meeting.
- To maintain confidentiality, I would ask that information provided in the focus group must be kept confidential by the focus group participants. Please do not discuss or share with others beyond this group, what is said in this meeting.
- The research team will handle confidentiality of all identifiable information (names, email and/or telephone numbers, voices).
- It is important for us to hear each other, contribute in the discussion. Please can one person speak at any one time.
- If it is at all possible, please could all mobile phones be switched off or turn to silent. I anticipate this focus group discussion to last for approximately 90 minutes.
- I would like to ask you to draw on all previous experiences and roles when considering your answers to these questions. We know that there are differing opinions between pharma and academia and would be interested to hear this difference in opinions from focus group members who have worked in both fields. We are also interested in opinions from any additional roles you may hold such as from working as part of an oversight committee.

Just to check if everyone happy to turn the recorder on.

1. **Turn on the recorder**

Could everyone please introduce themselves and their roles.

Let’s begin:

**Questions**

| **This section of the discussion is aimed specifically at better understanding the motivations behind current practice when selecting randomisation methods.** |
| --- |
| 1. **How do you currently select a randomisation method? (20 mins)**   For instance:   - Is there a standard preferred method?   - Does clinical area affect this?   - Or organisation policy? - What roles are involved in this decision process?   - Just statisticians or does IT/ the trial team have input into this - Do unit resources affect this choice?   - Would staffing issues/expertise lead to less complex method selection? - Are there specific circumstances where each method is used?   - For example does study design affect this choice?     - Sample size     - Number/type of prognostic factors     - Number of centres     - Parallel or multiarm     - Individually or cluster randomised     - Blinded or unblinded (who is blinded) |
| 1. **Do you have any specific opinions with respect to certain methods? (10 mins)**   For instance:   - Are simpler methods more effective? - Do you prefer to include balancing factors? - What are your views on Response Adaptive/Bayesian Adaptive methods?   - (Response adaptive methods are a group of methods where the ratio of participants assigned to each of the treatment groups is adjusted based on interim data obtained from the trial.) |
|  |
| **In this section we want to discuss which features of a randomisation method you consider important when monitoring the performance of randomisation methods – For example in a small trial where gender is known to affect the outcome you may wish to ensure balance in gender between groups.** |
|  |
| Break to show slides on balance and predictability and explain the tradeoff between them |
|  |
| 1. **Which method features do you think are important? (10 - 15 mins)**  - We have given balance and predictability as examples – can you think of any others   - Ease of use   - Ethical considerations   - Unit resources (if mentioned above as possibly affecting method selection |
| 1. **Why do you think that these features are important? (10-15 mins)**  - E.g., Balance may be considered important for:   - Statistical efficiency   - Perceived credibility of the trial findings   - Logistical reasons (management of IMP supply/intervention delivery) - **Why do you not consider these features important if you think that they are not?**   - E.g., Imbalance may be adjusted for within an analysis |
| 1. **How do you quantify/monitor these features within your own trials? (10-15 mins)**  - Do you only check sequences before the trial begins or do you monitor throughout? - Have you had any thoughts on other ways that you could measure these features?   - How can we account for the change in predictability given study design? |
