# Supplementary material for: Choosing and evaluating randomisation methods in clinical trials: a qualitative study
Source: Trials. 2024 Mar 20;25:199. doi: 10.1186/s13063-024-08005-z (PMC10953118; doi:10.1186/s13063-024-08005-z)
Supplement: Supplementary file 3 — Additional file 3. [file 13063_2024_8005_MOESM3_ESM.docx]

Additional file 3: The consolidated criteria for reporting qualitative research (COREQ)

| **Domain 1: Research team and reflexivity** |  | Location in manuscript (Section, page no.) |
| --- | --- | --- |
| **Personal Characteristics** | | |
| 1. Interviewer/facilitator Which author/s conducted the interview or focus group? | CB, CP, RO | Methods, Page 4 |
| 2. Credentials  What were the researcher’s credentials? E.g. PhD, MD | CP – PhD  RO – PhD  CB – MSc | - |
| 3. Occupation  What was their occupation at the time of the study? | CB – Medical statistician / PhD student  CP - Medical statistician  RO - Medical statistician | Methods, Page 4 |
| 4. Gender Was the researcher male or female? | One female and two male. | - |
| 5. Experience and training  What experience or training did the researcher have? | CP, RO and CB all come from a statistics background and so have experience in the subject matter. CB attended training on qualitative methods and two qualitative experts KS and MI advised on the design of the topic guide and the most appropriate analysis method to use considering our aims and data. | Methods, Page 4 |
| **Relationship with participants** | | |
| 6. Relationship established  Was a relationship established prior to study commencement? | Yes | - |
| 7. Participant knowledge of the interviewer  What did the participants know about the researcher? e.g. personal goals, reasons for doing the research | Participants were briefed on the purpose of the study and understood that aim was to explore their current practice when selecting a randomisation method and views of the different methods. Participants reviewed the participant information sheet and provided their informed consent before starting the focus group. | Methods, Page 4 |
| 8. Interviewer characteristics What characteristics were reported about the interviewer/facilitator? e.g. Bias, assumptions, reasons and interests in the research topic | A limitation of this study is the fact that coding was only completed by one researcher, CB. We acknowledge that this does have the potential to introduce bias, however feel that the effect would be limited. Focus groups were always attended by at least two researchers working on the project. During focus groups we encouraged open and honest discussions, which is evidenced by the contrasting opinions presented in the data and in the analysis. | Discussion, Page 24 |
| **Domain 2: study design** | | |
| **Theoretical framework** | | |
| 9. Methodological orientation and Theory  What methodological orientation was stated to underpin the study? e.g. grounded theory, discourse analysis, ethnography, phenomenology, content analysis | A deductive approach with inductive elements within a framework analysis was used. | Methods, Page 7 |
| Participant selection | | |
| 10. Sampling  How were participants selected? e.g. | Recruited via email | Methods, Page 4 |
| 11. Method of approach How were participants approached? e.g. face-to-face, telephone, mail, email | Email | Methods, Page 4 |
| 12. Sample size How many participants were in the study? | 25 | Results, Page 7 |
| 13. Non-participation How many people refused to participate or dropped out? Reasons? | Thirty one researchers responded initially. Of those, twenty five participated in the study, the other six did not respond when invited to the focus groups. | Results, Page 7 |
| **Setting** | | |
| 14. Setting of data collection  Where was the data collected? e.g. home, clinic, workplace | Data was collected via Teams platform. | Methods, Page 5 |
| 15. Presence of non-participants  Was anyone else present besides the participants and researchers? | No |  |
| 16. Description of sample  What are the important characteristics of the sample? e.g. demographic data, date |  | Table 1, Page 8 |
| **Data collection** | | |
| 17. Interview guide Were questions, prompts, guides provided by the authors? Was it pilot tested? | We used a topic guide to facilitate focus group discussions. (Additional File 1) | Methods, Page 5 |
| 18. Repeat interviews  Were repeat interviews carried out? If yes, how many? | No | - |
| 19. Audio/visual recording  Did the research use audio or visual recording to collect the data? | The focus groups were video and audio recorded. | Methods, Page 5 |
| 20. Field notes  Were field notes made during and/or after the interview or focus group? | Field notes were also used to record discussions and agreement during FGs. | - |
| 21. Duration What was the duration of the interviews or focus group? | The focus groups duration was between 75- 115 minutes | - |
| 22. Data saturation  Was data saturation discussed? | No | - |
| 23. Transcripts returned Were transcripts returned to participants for comment and/or correction? | No | - |
| **Domain 3: analysis and findings** | | |
| **Data analysis** | | |
| 24. Number of data coders  How many data coders coded the data? | One | Methods, Page 7 |
| 25. Description of the coding tree  Did authors provide a description of the coding tree? | No | - |
| 26. Derivation of themes  Were themes identified in advance or derived from the data? | Topics were predetermined (Additional file 1) but themes were derived from the data | Methods, Page 5  Results, Page 8 |
| 27. Software What software, if applicable, was used to manage the data? | Nvivo | Methods (Analysis), Page 7 |
| 28. Participant checking  Did participants provide feedback on the findings? | No | - |
| **Reporting** | | |
| 29. Quotations presented Were participant quotations presented to illustrate the themes / findings? Was each  quotation identified? e.g. participant number | Yes, specific comments were supported with direct quotes attributed to anonymised participant by role | Results, Pages 8-20 |
| 30. Data and findings consistent  Was there consistency between the data presented and the findings? | Yes | - |
| 31. Clarity of major themes  Were major themes clearly presented in the findings? | Yes | - |
| 32. Clarity of minor themes  Is there a description of diverse cases or discussion of minor themes? | Some | Results, Pages 4-148-20 |
