# Supplementary material for: Choosing and evaluating randomisation methods in clinical trials: a qualitative study
Source: Trials. 2024 Mar 20;25:199. doi: 10.1186/s13063-024-08005-z (PMC10953118; doi:10.1186/s13063-024-08005-z)
Supplement: Supplementary file 4 — Additional file 4: Table S1. Quotes on institutional standards. Table S2. Additional study design features mentioned by researchers. Table S3. Quotes around the definition of predictability. [file 13063_2024_8005_MOESM4_ESM.docx]

**Supplementary material**

Table S1 – Quotes on institutional standards

| **Quote (researcher)** |
| --- |
| *“…you know is that there's like a default institutional sort of preference. The last place I worked that was also a default institutional preference as well for minimisation.” (Programmer 3)* |
| *The default position now in [institution] is minimisation and therefore you know, it's almost like we justify not minimising prior. (Statistician 1)* |
| *Now the default position at [institution] for many years has been the opposite. It has been the stratified blocks (Statistician 15)* |
| *Our standard is stratified blocked for very similar reasons. (Statistician 13)* |
| *I think we tend to where I work at least, go for kind of stratified block randomisation schemes unless you know it's uhm, you’re randomising a small number of clusters, in which case some people seem to quite like minimisation. (Statistician 5)* |
| *“…so far I've come across only stratified randomisation.” (Statistician 16)* |
| *“Our default is to go simple.” (Statistician 11)* |
| *“I think pretty much all the trials in our units have used stratification with permuted blocks, even the large ones.” (Statistician 17)* |
| *“I see minimisation the vast majority of times, and if I'm ever asked to check a simulation, usually it's of minimisation as well. So that certainly seems to be the favourite for trials being run out of [institution]” (Statistician 18)* |
| *“So the randomisation is done on Redcap and that tends to be basically, as simple as we can make it.” (Statistician 19)* |

Table S2 – Additional study design features mentioned by researchers

| **Trial design feature** | **Quote (researcher)** |
| --- | --- |
| The intervention being delivered, emergency situations may not have time for more complex randomisation. | *“Delivery. I mean, I once did an emergency department trial, but it was only in three centres and each centre had their own list.” (Statistician 3)*  *“We came to the conclusion that was actually the only way, you know, from the practical perspective that randomisation could actually work. There's no phone line. There's nothing like that. Just grab the pack and off you go” (Statistician 1)* |
| If the trial is multi centred, 6 participants discussed inclusion of centre on the randomisation to avoid drug supply issues as a factor in which method they would select. | *“Sometimes drug supply means that you're forced to stratify by site, not through any particular biological reason or scientific reason, It's just practicality.” (Statistician 7)* |
| The level of validation the system requires, especially prevalent in CTIMPS. | *“we've done our own internal validation, but it might not meet the standardized standards for things like say a CTIMPs trial…I think maybe the choice of randomisation might be informed by things like the nature of the intervention, and the level of validation that you need to have” (Statistician 4)* |
| The cost involved in the randomisation method and time given to make decisions | *“I guess there might be an element of cost that we need to consider because obviously minimisation would cost more” (Statistician 13)* |
| Trial changes such as if arms are likely to enter and leave the trial – more adaptive designs may be needed | *“So for example, if you've got an adaptive design whereby you know there's some unknown new arms that might appear, and other ones might drop out and you've got… That has a real, quite significant tangible impact on how you should go about implementing it” (Programmer 3)* |

Table S3 – Quotes around the definition of predictability

| **Quote (researcher)** |
| --- |
| *“…are we checking for whether people have tried to predict it and therefore influence who they've recruited…?” (Statistician 8)* |
| *“And In the real world, does anybody really try and predict what's coming next?” (Statistician 9)* |
| *“People may try to subvert randomisation. But do they actually do it right anyway?” (Statistician 1)* |
| *“Whether any clinicians actually have the time or energy or are motivated enough to do that as another question.” (Statistician 5)* |
| *“We don't know how much people keep logs, are try to subvert systems and that is a continual trade off that we don't know what we're trading off.” (Statistician 11)* |
